# Radiation therapy and IRreversible electroporation for intermediate risk prostate cancer (RTIRE)

**DOI:** 10.1186/s12894-024-01506-8

**Published:** 2024-07-25

**Authors:** Marshall Diven, Karla Ballman, Ariel Marciscano, Christopher Barbieri, Jennifer Piscopo, Shu Wang, Himanshu Nagar, Timothy McClure

**Affiliations:** 1 Department of Radiation Oncology, NewYork-Presbyterian Brooklyn Methodist Hospital, Brooklyn, NY USA; 2 Division of Clinical Trials and Biostatistics, Department of Quantitative Health Sciences, Mayo Clinic, Rochester, MN USA; 3 Department of Radiation Oncology, Massachusetts General Hospital, Boston, MA USA; 4 Department of Urology, Weill Cornell Medicine, New York, NY USA; 5 Department of Urology, Weill Cornell Medicine, New York, NY USA; 6 Department of Urology, Weill Cornell Medicine, New York, NY USA; 7 Department of Radiation Oncology, Memorial Sloan Kettering Cancer Center, New York, NY USA; 8 Department of Urology, Weill Cornell Medicine, New York, NY USA

**Keywords:** Prostate cancer, Electroporation, Radiation, SBRT, Focal therapy, IRE

## Abstract

**Introduction:**

Radiation Therapy and IRreversible Electroporation for Intermediate Risk Prostate Cancer (RTIRE) is a phase II clinical trial testing combination of radiation therapy and irreversible electroporation for intermediate risk prostate cancer

**Background:**

PCa is the most common non-cutaneous cancer in men and the second leading cause of cancer death in men. PCa treatment is associated with long term side effects including urinary, sexual, and bowel dysfunction. Management of PCa is based on risk stratification to prevent its overtreatment and associated treatment-related toxicity. There is increasing interest in novel treatment strategies, such as focal therapy, to minimize treatment associated morbidity. Focal therapy alone has yet to be included in mainstream guidelines, given ongoing concerns with potentially higher risk of recurrence. We hypothesize combining focal therapy with whole gland, reduced dose radiotherapy will provide acceptable oncologic efficacy with minimal treatment associated morbidity. RTIRE is a phase II single institution, investigator-initiated study combining a local ablative technique though local irreversible electroporation (IRE) with MR guided RT (MRgRT) to treat the entire prostate. The goal is to provide excellent oncologic outcomes and minimize treatment related side effects through leveraging benefits of locally ablative therapy with established radiation treatment techniques.

**Methods:**

A total of 42 men with intermediate risk PCa per NCCN guidelines and focal grade group (GG) 2 or 3, Gleason Score (GS) 3 + 4 or GS 4 + 3, cancer in an MRI target will be enrolled. Patients with MRI visible foci of GG2/GG3 will undergo focal therapy with IRE of this lesion. Following successful focal therapy, patients will then undergo a course of reduced dose, whole gland MRgRT with either 32.5 Gy in 5 Fractions or 22 Gy in 2 fractions. The primary objective of the study is to determine safety. Secondary outcomes include evaluation of oncologic efficacy (as measured by the proportion of patients free of clinically significant cancer as defined as > Grade Group 1 at 1-year follow-up biopsy), imaging characteristics of patients pre and post RTIRE, impact on quality of life (QoL), and PSA kinetics.

**Discussion:**

Combining IRE with a reduced dose radiotherapy may offer a new treatment paradigm for PCa by both reducing treatment effects of full dose radiotherapy and minimizing the risk of recurrence observed with focal therapy.

**Trial Registration:**

Clinicaltrials.gov identifier: NCT05345444. Date of registration: April 25, 2022. Protocol Version: 6.0, July 7, 2023.

**Supplementary Information:**

The online version contains supplementary material available at 10.1186/s12894-024-01506-8.

## Background

PCa is the most common non-cutaneous cancer in men and the second leading cause of cancer death in men. In the US in 2023, PCa is estimated to account for 29% of new cancer diagnoses and 11% of cancer related deaths [1]. The current treatment paradigm for men with newly diagnosed prostate typically begins with initial disease risk stratification based on a multitude of factors specific to the patient and their cancer. A commonly employed stratification framework is published in National Comprehensive Cancer Network PCa guidelines. Recommendations regarding additional work up and definitive treatment recommendations are based on disease risk in the context of predicted life expectancy. Initial risk ranges from very low to high risk depending on stage, PSA as well as pathology from biopsy. Standard of care treatment options for men with favorable intermediate risk disease include active surveillance, radiotherapy or prostatectomy [2]. Individualized oncologic treatment takes into account patient factors, expected outcomes from the aforementioned treatment strategies while balancing risks, benefits and expected acute/ long term side effects and tailoring recommendations through shared decision-making process.

Low risk PCa patients, who are unlikely to benefit from treatment, as treatment does not outweigh treated associated toxicity or risks, are placed on active surveillance (AS). AS consists of repeat laboratory analysis, imaging, and prostate biopsy at variable intervals. Patients with localized intermediate or high-risk PCa are offered definitive treatment: surgery or radiation therapy. Thus, PCa patients with low risk or clinically insignificant PCa do not need treatment whereas those with intermediate/high risk PCa or csPCa do need treatment [3]. Unfortunately, both surgery and radiation therapy are not without significant treatment-related toxicity including sexual and urinary dysfunction – which impacts not only PCa patients but also their partners. There is significant interest among physicians and patients for treatment options that minimize these side effects, but also deliver definitive cancer treatment, i.e., focal therapy [4].

## Methods/Design

**Fig. 1:**
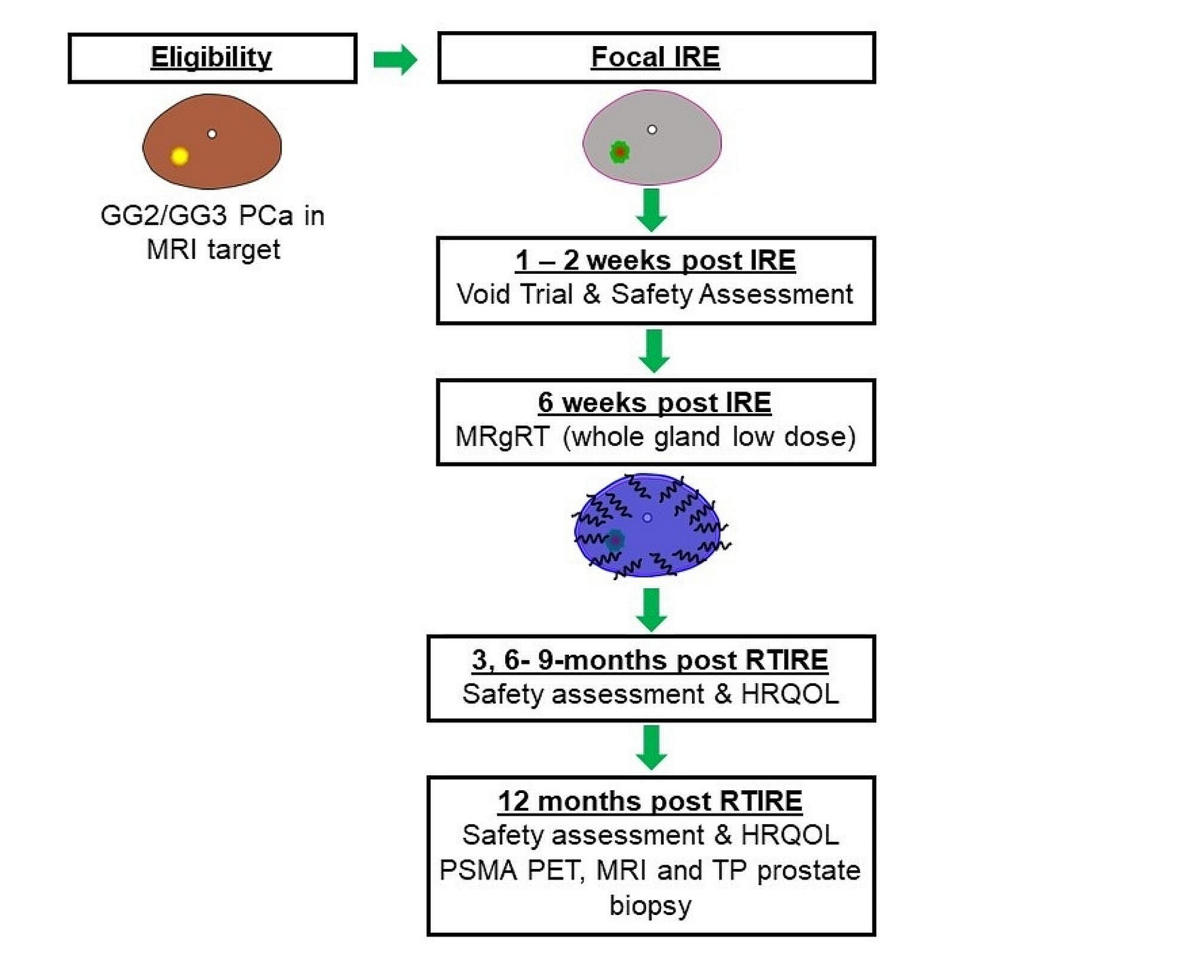
Treatment Workflow

This is a single site phase II trial for subjects with intermediate risk PCa with a focal GG2 or GG3 lesion with MRI target who will undergo IRE followed by SBRT with workflow shown in Fig. 1. We hypothesize that the combined therapy will be feasible and safe to perform with low morbidity and enhanced oncologic efficacy. The initial feasibility study (*N* = 10) is to determine the ability to perform IRE followed by SBRT. The primary endpoint is feasibility which is defined at 80% of subjects (8 subjects) assessed at 12-week post-IRE/6 weeks post-SBRT within 1 year from first subject enrollment. If the feasibility is met, the trial will then accrue an additional 32 patients for inclusion in the phase II trial.

**Fig. 2:**
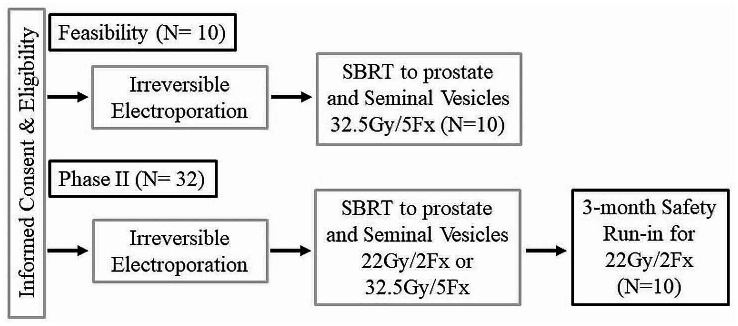
Trial Schema

The phase II component has a safety run-in cohort of patients. The first 10 patients after the feasibility portion will be treated with 2 fractions of RT. If at the 3-month follow-up, 3 or more patients have reported a grade 3 or higher AE, the 2-fraction schedule for RT will be deemed not tolerable and the remaining patients (*N* = 22) will be treated with the 5 fraction of RT schedule. All patients (*N* = 10 feasibility, *N* = 10 safety run-in, and *N* = 22) will be evaluable for the Phase II component. The general trial schema for the feasibility and phase II portion of the study are shown in Fig. 2. The co-primary endpoints are safety and oncologic efficacy evaluated as the proportion of patients who remain csPCa-free (> Grade Group 1) at the one-year follow-up. Note that this will be determined by the number of patients who are csPCA free divided by the total number of patients. If a patient does not have a one-year follow-up, they will be classified as a failure (i.e., are not cancer free). Additionally, patients with GG1 disease on 12-month post treatment biopsy will not be classified as a failure.

Secondary objectives will include determining short-term post-treatment safety profile, oncologic efficacy of RTIRE at 12 months. Determine the impact of RTIRE on health-related quality of life (HRQOL), post-RTIRE treatment prostate-specific antigen (PSA) kinetics including time to PSA nadir and post-nadir PSA stability, assess the effectiveness of therapy by post-treatment multiparametric magnetic resonance imaging (mpMRI) to evaluate the area of necrosis and presence of residual tissue, assess the effectiveness of therapy by recording the rates of biochemical and clinical progression and the need for secondary or adjuvant treatment following therapy. A full list of primary, secondary, and exploratory objectives as well as full inclusion and exclusion criteria are available in the attached supplemental file or on the corresponding trial page found on clinicaltrials.gov website.

## Patient selection, study enrollment, and randomization and blinding

Subjects ≥ 18 years of age with a new diagnosis of intermediate risk PCa and a focal GG2 or GG3 lesion in MRI target who meet the full inclusion and exclusion criteria will be notified of their eligibility for participation in this clinical trial. Subjects who agree to participate in the trial will sign the approved informed consent form and will be provided with a copy of the signed document. Subjects will be registered within the Weill Research Gateway-Clinical Trials as per the standard operating procedure for Subject Registration. Subjects will then undergo a screening visit to ensure all aspects of screening are complete as shown in Table 1.

**Table 1:** Schedule of Trial Events

Study Procedures	Screening	IRE	Post-IRE 1-2wk FUP	Last Day RT	3,6,9-mo FUP	12-mo FUP	24-mo FUP
Informed Consent	X						
Demographics/ MH	X						
Physical Exam	X		X	X	X	X	X
VS, Height, weight	X		X	X	X	X	X
PSA	X				X	X	X
Pelvic MRI	X*					X	
PSMA PET	X*					X	
EPIC-CP	X			X	X	X	X
IPSS	X			X	X	X	X
IIEF-5	X			X	X	X	X
Tissue Biopsy (Research)		X					
Research samples (Blood, Urine)	X			X		X	
Adverse Events (CTCAE v5.0)	X	X	X	X	X	X	X
Post-Treatment Biopsy (+/- 1 month)	X					X	

*Imaging completed ≤ 9 months acceptable for enrollment

IRE Irreversible electroporation, FUP follow up visit, mo months, MH medical history, PSMA PET prostate-specific membrane antigen positron emission tomography, EPIC-CP Expanded PCa Index Composite for Clinical Practice, IPSS International Prostate Symptom Score, CTCAE v5.0 Common Terminology Criteria for Adverse Events version 5.0

All subjects will undergo a transperineal 14-core template biopsy using UroNav’s ultrasound/MRI fusion technology to confirm outside diagnosis and/or establish eligibility wherein patients have 1 identified area of csPCa with acceptance of non csPCa in other cores. Additional targeted biopsies of lesions seen on mpMRI or PSMA-PET/CT will also be performed but patients at minimum will have a baseline 14-core systematic biopsy at baseline. Patients must have pelvic MRI and PSMA PET-CT within 9 months of enrollment on the study and will have updated H&P including full medical history, vital signs, PSA, as well as baseline EPIC-CP, IPSS and IIEF-5 questionnaire completed at screening visit. Blood and urine samples taken at the initial screening visit will be collected as well. Patients will be scheduled for their outpatient IRE procedure at a suitable future scheduled date. There will be no randomization performed after enrollment and neither clinicians, subjects nor data analyzers blinded to the assigned treatment.

## Interventions

### Irreversible electroporation

Following completion of informed consent and enrollment on the study, subjects will undergo IRE procedure targeting the focal csPCa performed by board certified Urologist with proficient prior training in the technique. Prior to NanoKnife treatment, subjects will receive an antibiotic of choice selected by the treating physician via intravenous infusion to reduce the chance of infection. A negative urine culture will be obtained prior to treatment. In the OR, subjects will be placed in the dorsal lithotomy position under sterile technique. The NanoKnife procedure will be carried out under general anesthesia. A Foley catheter will be placed to aid in draining the bladder during treatment.

The area of the prostate that was positive for cancer based on the transperineal prostate biopsy will be targeted for ablation via the NanoKnife System. An MRI/TRUS fusion device probe will be placed in the rectum and the prostate will be visualized in both sagittal and axial views. The ultrasound grid which was used during the mapping biopsy will be oriented using anatomical landmarks and used to identify the location of the positive biopsy cores. The NanoKnife Single Electrode Probes will be percutaneously inserted into the prostate through the perineum using UroNav MRI/TRUS fusion system as guidance. The location of the probes will be documented via ultrasound imaging.

After placement of the NanoKnife probes in the prostate and immediately prior to NanoKnife treatment, a nondepolarizing neuromuscular blocking agent will be administered to reduce skeletal muscle contraction which is associated with the use of the NanoKnife System. The pulse treatment dose will be determined using standard protocol and a pre-treatment checklist will be performed prior to ablation. Post ablation assessment will be performed by assessing changes in voltage parameters during treatment and after confirmation of adequate ablation the probes will be removed. An immediate post ablation MRI will also be assessed to determine the effectiveness of ablation. The Foley catheter will be left in place after the procedure and removed at the discretion of the treating physician. Any adverse events (AEs) will be recorded on the AE CRF. Patients will be discharged from the hospital with a foley catheter and scheduled for a void trial prior to removal. The void trial will include a post void residual to confirm safety for catheter removal. Patients will follow up with treating physician in 1–2 weeks post ablation and will be eligible to undergo RT planning no sooner than 6 weeks post IRE.

### Radiotherapy

After consent, eligibility verification, and IRE patients will undergo CT/MRI simulation and radiotherapy planning. Patients will receive treatment to the prostate + seminal vesicles to a dose of 32.5 Gy in 5 fractions or 22 Gy in 2 fractions. The rationale behind the selected dose relates to an estimated a/b ratio of 2.7 for PCa supported by ultra-hypofractionation trials and a large meta-analysis [5, 6]. With a lower α/β value, PCa should have an improved therapeutic ratio with ultra-hypofractionation. Furthermore, if the α/β formalism and assumed values for PCa are correct and one maintains a constant biologically effective dose for normal tissues, there is the potential for increased tumor control with ultra-hypofractionation in this setting. The dose selected for this trial is less than the therapeutic dose when radiation alone is used for intact PCa (7–8 Gy x 5 fractions or 12.5–14 Gy x 2 fractions) but has a biological effective dose of > 100 Gy based on the α/β value of 2.7 Gy.

Full details regarding radiation therapy treatment planning/simulation set-up, contouring, PTV and OAR prescription dose constraints, adaptive MR-Linac planning can be found in Supplemental File 1.

### Follow up and trial procedures

Subjects will be seen on their final day of radiotherapy at which time blood and urine samples will be collected for research purposes as outlined in treatment protocol and previously discussed at time of informed consent/enrollment. Subjects will also complete EPIC-CP, IPSS and IIEF-5 questionnaires on the final day of RT and evaluated for any AEs per CTCAE v5.0 definition. They will be informed regarding expected side effects following treatment and will be counseled to contact treating physician/medical provider for any concerning side effects following treatment.

A proportion of patients undergoing prostate radiotherapy can expect an increase in urinary frequency or urgency. If this becomes bothersome to the patient, medication to alleviate symptoms can be prescribed at the discretion of the treating radiation oncologist and documented in patient chart. Serious bowel symptoms during time of prostate radiotherapy are rare. If patients develop rectal urgency, tenesmus or diarrhea, medication to alleviate symptoms can be prescribed at the discretion of the treating radiation oncologist and documented in patient chart.

Subjects will be scheduled for post RT follow up appointments at 3, 6, 9, 12 and 24 months. At 3, 6, 9, 12 and 24 month follow up visits all patients should undergo physical exam including vital signs, height, weight, PSA, repeat EPIC-CP, IPSS, IIEF-5 and screened for adverse events.

At the 12 month follow up appointment, patients will undergo pelvic MRI, PSMA PET-CT, collection of blood and urine specimen as well as repeat transperineal 14-core template biopsy (Additional biopsies of targeted lesion/ and new PSMA PET/CT or mpMRI identified lesions will also be performed) using UroNav’s ultrasound/MRI fusion technology. These prostate biopsy specimen will be analyzed for oncologic effectiveness as part of the primary objective of the study. Planned trial procedures are outlined in Table 1.

### Adverse events

Adverse event (AE) monitoring and reporting is a routine part of every clinical trial. CTCAE v5.0 will be utilized to grade any potential adverse events in relation to the trial at specified time points and any unscheduled time points while patient is on the study. All adverse events will be recorded on a subject specific AE log which will be maintained by the research staff and kept in the subject’s research chart. All AEs and SAEs occurring on this study will be reported to the Institutional review board (IRB) of Weil Cornell Medicine according to the IRB policy and as outlined in the current approved study protocol. All SAEs and AEs reported during this study will be followed until resolution or until the investigator confirms that the AE/SAE has stabilized, and no more follow-up is required.

### Data management and safety monitoring

REDCap will be used to collect and maintain all data related to the study including details of treatment, toxicity, efficacy, and AE data for all enrolled subjects as outlined in the trial protocol and per WCM IRB approved protocols. Security measures to protect patient data include firewall technology, database level security with minimum necessary privileges is routinely employed. Data is backed up periodically per institutional standard operating procedures and is stored for at least 5 years following the termination of the study. The Weil Cornell Medicine Data Safety Monitoring board serves as the central monitoring body and operates in concordance with the guidelines in the 2001 National Cancer Institute-approved data and safety monitoring plan for Weill Cornell Medicine Meyer Cancer Center. Additional information regarding data management and safety monitoring can be obtained online via WCM IRB website.

### Statistical analysis

#### Sample size and accrual

For the phase II portion of the trial, it is assumed that the historical control value for the. proportion of patients who are cancer free at 1-year follow-up is 0.80 for the standard of care comparison.

The trial is designed to detect an increase in the proportion of patients who are csPCa free at 1-year follow-up to 0.95 (increase of 0.15). An exact binomial test with a 10% one-sided significance level will have 94.3% power to detect the difference between the null hypothesis proportion of 0.8 and the alternative proportion of 0.95 when the sample size is 42. All subjects who received any protocol treatment will be included in the evaluation of adverse events from the time of their first treatment with RTIRE. All subjects included in the study will be assessed for treatment efficacy if they have received any protocol IRE and radiation treatments.

The primary analysis for the Phase 2 portion will include the patients that were part of the feasibility evaluation (*N* = 10), the patients who were part of the safety run-in for the 2 fraction RT schedule (*N* = 10), and the additional patients who were accrued after the run-in phase (*N* = 22) for a total of *N* = 42. The analysis will use a one-sided exact binomial test to determine if the observed proportion of patients who are cancer free at the one-year follow-up is greater than 0.80. In addition, the proportion of patients who are cancer free at the one-year follow-up will be estimated with a binomial point estimate and 80% binomial confidence interval. We will also generate and report the 95% confidence interval since this is more familiar to the research community.

The maximum grade of a specific AE experienced by a patient will be used. Unique AEs and the grade will be summarized as frequency and relative frequency. The proportion of patients who experience a grade 3 or higher (and grade 4 or higher) will be estimated with a binomial point estimate and corresponding 95% confidence interval. The QOL measures will be plotted over time for each patient and an average curve will be superimposed. The change in QOL from baseline will be summarized at each timepoint by the mean, standard deviation, median, minimum, and maximum value. PSA values will also be plotted over time for each patient with an average curve superimposed. The proportion of patients who experience a biochemical recurrence by one year will be estimated with a binomial point estimate and 95% confidence interval. The time to biochemical recurrence will be estimated with a Kaplan-Meier estimator.

## Discussion

Focal therapy is based on the index lesion theory which is centered upon the fact that clinically insignificant PCa can be monitored and surveilled whereas clinically significant prostate cancer (csPCa) needs to be treated and can be treated focally, with an ablative technique. By focally ablating csPCa, a patient’s risk can be moved from an intermediate or high-risk category, down to low risk – thereby avoiding definitive treatment and being put back on AS. An important advantage of focal therapy is that it results in decreased side effects, because only a small area of the prostate is treated, thereby minimizing damage to surrounding muscles and nerves. As a result, sexual and urinary dysfunction side effects are less severe after treatment compared to prostatectomy.

Major limitations to focal therapy exist. The recurrence of cancer after focal therapy in both the ablation zone and in the non-ablated zone is significant. A phase II study, prospective demonstrated a recurrence of csPCa (as defined by GG > 1) after focal therapy was 40% at 2 years [7]. Thus patients undergoing focal therapy require close observation with repeat laboratory analysis, imaging, and biopsy. Despite these limitations with focal therapy, there remains strong interest in new treatment paradigms that provide cancer treatment with minimal treatment side effects.

Common side effects related to definitive treatment of localized PCa usually involve genitourinary (GU), sexual reproductive or gastrointestinal (GI) systems. The expected side effect profiles vary depending on the definitive treatment modality used as well as baseline patient function in each of those domains prior to treatment. Hoffman et al. published patient reported outcomes through 5 years for PCa patients undergoing active surveillance, surgery, brachytherapy or external beam radiation therapy (EBRT) +/- ADT and found that by 5 years most urinary, bowel, sexual differences attenuated. Despite stabilization of side effect profiles in these domains, the authors suggest clinically meaningful worse urinary incontinence remains for some men undergoing prostatectomy compared to EBRT or surveillance [3]. 

Urinary incontinence is a common urinary symptom for patients undergoing prostatectomy. In a prospective cohort study, Chen et al. report that 34.3% of men with normal baseline urinary control maintain normal control at 2 years following prostatectomy. This is in comparison to 73% for men undergoing EBRT and 72% for those in the active surveillance cohort. In this same study, EBRT was associated with an acute increase in urinary obstruction and irritative symptoms for patients with normal baseline function, reaching statistical significance at 3 months though gradually returning to baseline by 12 month follow up. This is in contrast to patients with poor baseline urinary obstruction and irritation with up to 75.7% of patients reporting improvement following prostatectomy at 2 years vs. 45.9% and 43.3% treated with EBRT vs. those undergoing AS, respectively [8]. Thus, RP might be a more suitable treatment option for some patients given the added benefit of RP for relieving severe bladder outlet obstruction [9]. 

Patient reported side effects were also reviewed as part of the ProtecT clinical trial and men treated with RT tend to have worse GI toxicities compared to those treated with RP with reports of 1 in 8 men experiencing long term toxicity. Recent trials examining late toxicities in patients undergoing stereotactic body radiotherapy (SBRT) with modern techniques suggest approximately 10% of patients may experience long term grade ≥ 2 GI toxicities and 0.4% grade ≥ 3 toxicities [10–12]. The recently published MIRAGE trial evaluated physican reported toxicities and patient reported symptoms for patients randomized to either MR-guided vs. CT-guided SBRT [13]. The results of the trial suggest that reduced treatment margin and lack of fiducial placement, allowed by intra-fraction MRI imaging for patients treated with MR-Linac, significantly reduced acute GI and GU toxic side effects compared to patients treated with CT guided radiotherapy. Grade ≥2 GU toxic effects were reported in 43.4% vs. 24.4% of patients treated with CT vs. MRI guided SBRT, respectively. There was no grade ≥ 2 GI toxic effects reported in patients treated with MRI guidance compared to 10% of patients undergoing SBRT with CT guidance. While there are some caveats regarding the interpretation and extrapolation of the results for this study as it relates to MRI vs. CT based SBRT, it serves as an excellent benchmark regarding acute toxicities and side effects in patients treated with modern SBRT techniques.

IRE is a local, minimally invasive ablative technique which uses physical placement of probes into the prostate tissue allowing for the application of pulsatile electrical currents to a focal target area to treat malignant tissue. These electrical currents lead to cell membrane destabilization causing alteration of the membrane shape and formation of nanopores which ultimately leads to irreversible damage and apoptosis through disruption of cellular osmotic balance [14]. The technique has been studied extensively in both animal models and in human subjects with promising oncologic and functional outcomes observed and well summarized in a review article by Ong et al. [15]. 

In many of the studies reporting on IRE, preservation of erectile function as well as maintenance of urinary continence are key factors assessed following treatment. Blazveski et al. report on a database which included 123 men treated with IRE and included EPIC questionnaire and AUA symptom score to assess QoL at baseline and following treatment. In that report they show excellent toxicity outcomes in the urinary and sexual domains with 98.8% (80/81) patients remaining pad-free and 98% (49/50) patients maintaining potency at 12 month follow up, respectively [16]. Reported oncologic outcomes from this particular study and others are promising with significant reduction in post treatment PSA and eradication of treated disease. However, a significant percentage of patients examined following treatment have in-field and/or out of field recurrences diagnosed on post-treatment biopsy necessitating additional local and/or whole gland treatment with RT or RP [7, 16–20]. Thus, for men with localized intermediate risk PCa confined to a portion of the prostate gland, current standards support whole gland treatment as routine standard of care.

Local ablative or focal therapy have yet to be established in management guidelines for PCa. This is due in large part to reported outcomes of studies on cryoablation or HIFU that are inferior to that of either surgery or definitive RT when looking at progression of disease in untreated prostatic tissue [21, 22]. In this context, we are investigating an approach where we are escalating treatment intensity to csPCA targetable lesions with IRE while giving the remainder of the intact prostate effective albeit reduced radiation dose. Our goal from this approach is to optimize oncologic and QOL outcomes related to treatment toxicity. RTIRE is a phase I/ II single arm, investigator-initiated study combining a local ablative technique though local irreversible electroporation (IRE) and SBRT to treat the entire prostate gland. The goal is to provide excellent oncologic outcomes and minimize treatment related side effects through leveraging benefits of locally ablative therapy with established radiation whole gland treatment techniques using image guided SBRT.

## Conclusions

The current standard of care definitive treatment for patients with newly diagnosed localized intermediate risk PCa involves whole gland treatment with either radical prostatectomy (RP) or definitive radiotherapy (RT). Balancing risks/side effects from intervention is paramount for navigating the informed decision-making process and informing patients on optimal management strategy based on available data. RTIRE is a trial designed to evaluate a potentially new paradigm of treatment for men with intermediate risk PCa and a focal csPCa targetable lesion. The proposed strategy utilizes a combined treatment approach with a focal targeted IRE procedure combined with dose reduced prostate SBRT. The goal is to provide effective oncologic treatment for patients while simultaneously minimizing definitive treatment related side effects seen with other treatment approaches.

### Electronic supplementary material

Below is the link to the electronic supplementary material.

Supplementary Material 1

## Data Availability

Due to the ongoing nature of this trial and with respect to the privacy of our human participants, any datasets generated and/or analyzed during the current study may be made available, at the discretion of the principal investigator, at an appropriate future date upon reasonable request. Any potential data generated and or analyzed and made available would be done so in accordance with procedures set forth by an appropriate regulatory body to ensure the privacy of the participants in this study.

## Abbreviations

PCa: Prostate cancer
IRE: Irreversible electroporation
MRgRT: Magnetic resonance-guided radiotherapy
NCCN: National Comprehensive Cancer Network
MRI: Magnetic resonance imaging
PSA: Prostate-specific antigen
AS: Active Surveillance
csPCa: Clinically significant prostate cancer
GU: Genitourinary
GI: Gastrointestinal
EBRT: External beam radiation therapy
ADT: Androgen deprivation therapy
SBRT: Stereotactic body radiation therapy
CT: Computed tomography
PET: Positron emission tomography
MR-LINAC: Magnetic resonance linear accelerator
QoL: Quality of life
EPIC: Expanded Prostate Cancer Index Composite
HRQOL: health-related quality of life
CTCAE: Common Terminology Criteria for Adverse Events
OAR: Organs-at-risk
ECOG: Eastern Cooperative Oncology Group
IPSS: International Prostatism Symptom Score
PSMA: Prostate-specific membrane antigen
PTV: Planning target volume
CTV: Clinical target volume
PRV: Planning organs-at-risk volume
AE: Adverse event
SAE: Serious adverse event
IRB: Institutional Review Board
REDCap: Research Electronic Data Capture
