# Supplementary material for: Radiation therapy and IRreversible electroporation for intermediate risk prostate cancer (RTIRE)
Source: BMC Urol. 2024 Jul 25;24:151. doi: 10.1186/s12894-024-01506-8 (PMC11271032; doi:10.1186/s12894-024-01506-8)
Supplement: Supplementary file 1 — Supplementary Material 1 [file 12894_2024_1506_MOESM1_ESM.docx]

**Supplemental File 1**

***Primary objectives***

Feasibility: Determine feasibility of combined IRE and SBRT. Feasibility is defined at 80% of subjects assessed at 12 weeks post-IRE and 6 weeks post-MRgRT within 1 year from first subject enrollment.

Phase II: Determine the potential efficacy of combined IRE and SBRT. The efficacy will be measured as the proportion of patients who are free of clinically significant cancer (> Grade Group 1) at the 1-year follow-up.

***Secondary Objectives***

1. Determine combined IRE and SBRT (RTIRE) short-term post-treatment safety profile.

2. Determine oncologic efficacy of RTIRE at 12-month biopsy.

3. Determine impact of RTIRE on health-related quality of life (HRQOL)

4. Determine post-RTIRE treatment prostate-specific antigen (PSA) kinetics including time to PSA nadir and post-nadir PSA stability.

5. Determine the effectiveness of therapy by post-treatment multiparametric magnetic resonance imaging (mpMRI) to evaluate the area of necrosis and presence of residual tissue.

6. To determine the effectiveness of therapy by recording the rates of biochemical and clinical progression and the need for adjuvant or salvage treatment following therapy.

***Exploratory objectives***

1. Imaging characteristic changes seen post ablation to assess for predictors of response.

2. Tissue, plasma and urine will be collected for analyses for predictors of cancer control and toxicity following RTIRE. Research samples will be collected at baseline, last day of radiation therapy, at 12-month post radiation therapy.

***Inclusion Criteria***

- Men aged ≥ 18
- ECOG 0 – 1
- Histologically confirmed intermediate risk PCa per NCCN guidelines
- Focal grade group 2 or 3 (GS 3+4 or GS 4+3) cancer in MRI target
- Gland size < 80 cc
- Ability to undergo IRE
- Ability to receive MRI-guided radiotherapy
- Ability to complete the HRQOL assessment surveys
- Willingness to undergo 12 month follow up biopsy

***Exclusion Criteria***

- Prior history of focal therapy
- Prior history of receiving pelvic radiotherapy
- Patient with metastatic PCa
- Patient with history of inflammatory bowel disease
- Inability to undergo general anesthesia
- Inability to be placed within the lithotomy position for a transperineal approach to both biopsy and treatment
- Patients with a prior or concurrent disease whose natural history or treatment does not have the potential to interfere with the safety or efficacy assessment of the investigational regimen are eligible for this trial. Note: Any patient with a cancer (other than keratinocyte carcinoma or carcinoma in situ or low-grade non-muscle invasive bladder cancer) who has been disease-free for less than 3 years must contact the Principal Investigator
- History of bladder neck or urethral stricture

**Radiotherapy**

***Treatment planning***

Immobilization of the hips and feet using a cradle should be considered. Each patient will be positioned in the supine position. Prone positioning for treatment is not permitted. Treatment planning CT/MRI will be performed with vac loc immobilization. Patients will be advised to drink 2-3 cups of water 30 minutes prior to the MRI/CT simulation to allow for a comfortably full bladder, as tolerated. An overly distended rectum can introduce a systematic positioning error that may increase the probability of missing the clinical target volume (CTV). Patients will be simulated with the rectum as empty as possible with <3 cm in the anterior-posterior dimension representing ideal set-up. Rectal balloons for planning and treatment are not permitted. Enema may be necessary for subjects and will be recommended at the discretion of the treating physician. Subjects will also have a comfortably full bladder.

***Contouring***

The prostate + seminal vesicles will be contoured as the clinical target volume (CTV). The planning target volume (PTV) expansion for the CTV will be 2 mm. The rectum will be drawn from the bottom of the ischial tuberosities to the sigmoid flexure. The bladder, bladder wall (bladder – 4 mm isotropic constriction), urethra (contoured with 10 mm brush on sagittal view), femoral heads, and penile bulb will also be contoured as normal structures.

***Dose/Treatment Planning Parameters***

PTV will be treated to the prescribed dose 32.5 Gy in 5 fractions or 22 Gy in 2 fractions. VPrescription Dose (volume of the PTV receiving Prescription Dose) should be ≥ 95% and not exceed 115% (hotspot). Urethra should be expanded with a 3 mm PRV and should not receive more than the prescription dose. Detailed constraints for individual OARs are included in Supplemental File 1.

***Adaptive Planning for Patients Treated on MR-Linac***

1. Prior to treatment, each patient will undergo set up MRI scan.

2. 2D shifts will be performed to align relevant anatomy (bladder wall, rectum, prostate +/- seminal vesicles).

3. Simulation contours will be rigid copied to set up scan and recontoured if CTV delineation changes.

4. Predict dose algorithm will determine if treatment dose parameters meeting planning dose parameters.

5. Patients will undergo adaptive planning if treatment dose parameters do not meet planning parameters AND per protocol planning parameters in **Table S1** & **Table S2**.

**Table S1.** 32.5Gy in 5 Fractions Organ at Risk (OAR) Constraints

| Structure | Dosimetric Parameter | Per Protocol |
| --- | --- | --- |
| Rectum | V33.11  V29.9  V28.4  V25.2  V15.9 | < 0.03 cc (optimal)  < 3 cc (optimal)  < 10% (mandatory)  < 20% (mandatory)  < 50% (mandatory) |
| Bladder | V33.11  V15.76 | < 0.03 cc (optimal)  <10% (mandatory) |
| Bladder Wall | V33  V15.9 | < 0.03 cc (optimal)  < 15 cc (optimal) |
| Urethra | V33.7 | < 0.03 cc (optimal) |
| Penile Bulb | V17.3 | < 3 cc (optimal) |
| Femoral Head (each) | V19.9  V15.6 | < 1 cc (optimal)  < 10 cc (optimal) |

**Table S2.** 22Gy in 2 Fractions Organ at Risk (OAR) Constraints

| Structure | Dosimetric Parameter | Per Protocol |
| --- | --- | --- |
| Rectum | V11.4  V15.5  V18.3  *Max Point Dose | < 7 cc (optimal)  < 4 cc (mandatory)  < 1 cc (mandatory)  < 23.8 Gy (optimal) |
| Bladder | V12.8  V18.3  *Max Point Dose | < 15 cc (mandatory) < 5 cc (mandatory)  < 23.8 Gy (optimal) |
| Bladder Wall | V12.8  V18.3  *Max Point Dose | < 15 cc (mandatory) < 5 cc (mandatory)  < 23.8 Gy (optimal) |
| Urethra | V23.7 | D10% (mandatory) |
| Penile Bulb | V17.1 | < 50% (optimal) |
| Femoral Head (each) | V12.3 | < 10 cc (optimal) |

**Irreversible Electroporation Procedure**

The NanoKnife System is the device that is used for IRE and is FDA approved for the surgical ablation of soft tissue. The NanoKnife System is composed of the NanoKnife Generator which contains the Power Unit, TPM Module, and user interfaces (foot switch, keyboard, touchpad, and screen), and the accessory NanoKnife Single Electrode Probes. The system may also be used in conjunction with an optional Pulse Trigger Sync Device while in ECG (electrocardiogram) synchronization mode. The NanoKnife System is composed of a software controlled low energy direct current (LEDC) generator which surgically ablates soft tissue and Single Electrode Probes.

With the NanoKnife System, electrical current is delivered between pairs of probes in a series of pulses. The waveform of the current is adjustable as determined by clinician chosen parameters. Up to six probes may be placed in an array within the tissue. The probes of the array are matched as pairs by the system. When probes are activated via a foot pedal, the scheduled current is delivered to tissue between subsequent pairs of probes. Soft tissue between the probes is ablated.

The NanoKnife Generator is a microprocessor controlled low energy direct current (LEDC) irreversible electroporation device. The NanoKnife Generator includes the following primary components: LCD Display, Console and Keyboard, Power Unit and Power Cord, and Double Footswitch/Foot Pedal. The generator can deliver up to 3000V of energy in a maximum of 100 pulses per pulse stream, which have a maximum pulse length of 100 μsec. The generator has user-controlled settings of voltage, pulse length, and number of pulses applied and is designed to be used in conjunction with the NanoKnife Single Electrode Probes supplied by AngioDynamics, Inc.

The performance specifications for the NanoKnife System are listed in the table below.

**Table S3.** NanoKnife System Specifications

| Number of Probe Outputs | 6 maximum |
| --- | --- |
| Number of Pulses for each Pair of  Electrodes | 10 to 100 |
| Pulse Amplitude | 500 to 3000V |
| Pulse Length | 20 - 100 μsec. |
| Pulse Interval, Un-sync | 90PPM, 670 ms/3.5 s every 10^th^ pulse |
| Maximum Energy per Pulse | (Nominal) 15 J |
| Maximum Current | 50 A |

The NanoKnife system is divided between a user interface and the implementation of user commands. The user interface consists of the computer console including the keyboard and touch pad, and the monitor touch screen. Through this interface, the system communicates with the user and the user inputs the desired functions to the system. This portion of the system is electrically isolated through optical isolation from the portion of the system which carries out the commands and physically isolated from the implementation of the system by the monitor mount. The connection of two Single Electrode Activation Probes is required to activate the Generator for a procedure to be executed. The NanoKnife System Electrode Probes comprised of the following:

1. Active electrode, length adjustable in 0.5 cm increments from 0 – 4 cm via the thumb

slide

2. Thumbslide

3. Insulation sleeve

4. 19 gauge needle with depth markers and echogenic needle tip

5. 10 foot connection cable

The NanoKnife Single Electrode Probes work in a two-pole operating mode where one is the positive voltage supply and the second is the negative return. Up to six NanoKnife Single Electrode Probes can be placed at a fixed distance apart in soft tissue to create several two pole electrode configurations. Depending on the size of the soft tissue area to be ablated, a maximum of six probes can be used in a procedure. The NanoKnife System will initially configure the probes into pairs in a default configuration; however, the pairings are editable by the user. NanoKnife Single Electrode Probes may also be repositioned after each procedure to cover a larger area at the user’s discretion per the Instructions For Use, and the System User Manual.

The basic principles of operation of the NanoKnife System consist of powering the unit on, entering patient and treatment parameters into the system interface, placement of electrode probes into the tissue that is targeted for ablation, activation of the system to deliver the desired treatment and removal of the probes from the tissue.
